# Ultrasonography guided fine needle aspiration cytology in patients with laryngo-hypopharyngeal lesions^☆^

**DOI:** 10.1016/j.bjorl.2018.11.005

**Published:** 2018-12-28

**Authors:** Lakshminarasimman Parasuraman, Chirom Amit Singh, Suresh C. Sharma, Alok Thakar

**Affiliations:** All India Institute of Medical Sciences, Department of Otorhinolaryngology, New Delhi, India

**Keywords:** Laryngeal cancer, Ultrasound guided transcutaneous fine needle aspiration cytology, Laryngeal pharyngeal lesions, Cytology, Câncer de laringe, PAFF-USGTC, Lesões laríngeo-hipofaríngeas, Citologia

## Abstract

**Introduction:**

Laryngeal lesions are usually evaluated by microlaryngoscopy/direct laryngoscopy under anaesthesia for disease mapping and tissue diagnosis. However patients with anticipated airway compromise due to laryngeal mass may require either a protective tracheotomy or emergency tracheotomy to secure the airway. To minimise risk of unplanned tracheotomy and expedite the diagnosis we performed ultrasound-guided transcutaneous fine needle aspiration cytology.

**Objective:**

To evaluate the feasibility and performance of ultrasound-guided transcutaneous fine needle aspiration cytology of suspicious/recurrent laryngo-hypopharyngeal masses.

**Methods:**

Fine needle aspiration cytology was performed under ultrasound guidance. Twenty- four patients were recruited, of which 17 had a pure laryngeal lesion; 6 patients had laryngo-pharyngeal, and one patient had a base tongue lesion with supra-glottis extension.

**Results:**

Out of 24 patients, 21 had positive cytology for squamous cell carcinoma, 2 patients had non-diagnostic cytology (atypical cells) and the other had inadequate tissue for definitive diagnosis. Patients with negative and inconclusive cytology underwent direct laryngoscopy biopsy, which was positive for squamous malignancy. All patients tolerated the procedure well and no adverse events were noted.

**Conclusion:**

Although direct laryngoscopy remains the standard of care in evaluation of laryngo-hypopharyngeal lesions, this pilot study has shown that ultrasound-guided transcutaneous fine needle aspiration cytology was feasible as an out-patient procedure, employing safe and sensitive technique enabling rapid diagnosis and avoiding the need for direct laryngoscopy under GA for tissue diagnosis.

## Resumo

**Introdução:**

As lesões laríngeas são geralmente avaliadas por microlaringoscopia/laringoscopia direta sob anestesia para mapeamento da doença e diagnóstico tecidual. No entanto, em pacientes com comprometimento prévio das vias aéreas devido à lesão laríngea, pode ser necessária uma traqueostomia protetora ou traqueostomia de emergência para assegurar as vias aéreas. Para minimizar o risco de uma traqueostomia não planejada e facilitar o diagnóstico, realizamos punção aspirativa por agulha fina guiada por ultrassonografia transcutânea.

**Objetivo:**

Avaliar a viabilidade e o desempenho da punção aspirativa por agulha fina guiada por ultrassonografia transcutânea em lesões laríngeo-hipofaríngeas suspeitas/recorrentes.

**Método:**

A punção aspirativa por agulha fina foi realizada sob orientação ultrassonográfica. Foram recrutados 24 pacientes, 17 com lesão laríngea apenas, 6 com lesão laríngeo-faríngea e um com lesão na base da língua com extensão supraglótica.

**Resultados:**

Dos 24 pacientes, 21 apresentaram citologia positiva para carcinoma espinocelular, 2 citologia não diagnóstica (células atípicas) e o outro tecido inadequado para o diagnóstico definitivo. Os pacientes com citologia negativa e inconclusiva foram submetidos à biópsia através de laringoscopia direta, que foi positiva para lesão maligna espinocelular. Todos os pacientes toleraram bem o procedimento e nenhum evento adverso foi observado.

**Conclusão:**

Embora a laringoscopia direta continue a ser o padrão de cuidado na avaliação das lesões laríngeo-hipofaríngeas, este estudo piloto demonstrou que a punção aspirativa por agulha fina guiada por ultrassonografia transcutânea é uma técnica viável, ambulatorial, segura e sensível, permite rápido diagnóstico e evita a necessidade de laringoscopia direta sob anestesia geral para diagnóstico tecidual.

## Introduction

Over the last two decades the technique of fine needle aspiration cytology has become the first line tissue diagnostic procedure for palpable head and neck mass lesions. For those clinically inaccessible lesions imaging modalities such as ultrasound, computed tomography, magnetic resonance imaging had been used to reach the targeted tissue for diagnosis.^1^

Laryngeal lesions are usually evaluated by microlaryngoscopy or direct laryngoscopy under anaesthesia for mapping the disease and to obtain tissue diagnosis. In a few cases, the bulk of the disease might be submucosal in nature, or in recurrent cases (i.e.) post treatment (radiotherapy/chemoradiotherapy) superficial mucosal biopsy may not be adequate for diagnosis.^2^

In a pilot study by Ansarin et al. Ultrasound-Guided Transcutaneous tru-cut Biopsy (USGTCB) was shown to be a useful technique for tissue diagnosis of bulky laryngeal masses in patients who are at risk for general anaesthesia.

Ultrasound-guided fine needle aspiration was performed in two patients for rapid tissue diagnosis who presented with supraglottic mass lesion with normal appearing surface mucosa. Cytology in both cases was diagnostic for squamous cell carcinoma.^3^

Preoperative factors like difficult intubation and anticipated compromised airway due to laryngeal mass after biopsy may necessitate either a protective or emergency tracheotomy to secure airway. The presence of tracheotomy implies a non-functional larynx in the majority of patients, making the option of organ preservation impractical.

Cervical spondylosis, multiple comorbidities and advanced age are a few patient factors that may at times make them unfit for evaluation under general anaesthesia.

The purpose of this study was to assess the feasibility and performance of USGTC-FNAC in untreated or previously treated suspicious laryngopharyngeal masses under local anaesthesia, as well as to evaluate its efficacy in obtaining a histological diagnosis.

## Methods

The study was conducted between January 2013 and June 2013. Patients with untreated or previously treated suspicious laryngeal pharyngeal masses were prospectively enrolled in the study after fulfilling the following inclusion criteria: (1) Advanced laryngeal and hypopharyngeal mass lesions (corresponding to T_3_ and T_4_ stage according to AJCC 7th edition); (2) At risk for tracheotomy (bulky laryngeal growth, signs indicating difficult intubation)^4^; (3) Contraindications for general anaesthesia.

The study was approved by the institutional ethical committee (IEC/NP-421/2012 & RP-12/2012) and written informed consent was obtained from all patients.

Those patients with early (T_1_, T_2_) lesions, massively calcified thyroid cartilage, vascular lesions, lesions not detected by ultrasound and those with abnormal coagulation profile were excluded from the study.

All patients were evaluated by history, physical examination, coagulation profile, examination by Hopkins (90 degree)/flexible fibreoptic laryngoscopy, and multi- slice spiral Contrast Enhanced Computed Tomography (CECT) of face and neck.

### Technique

**Figure 1:**
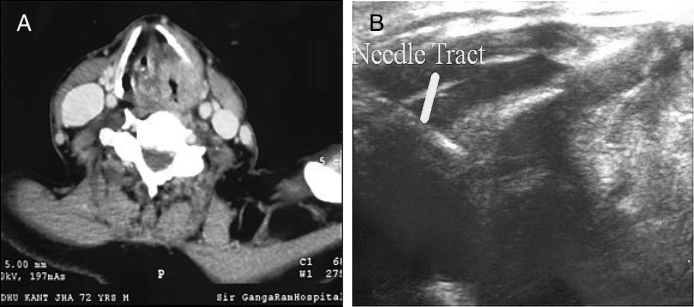
(A) 72 years/Male patient with suspected carcinoma larynx: CECT scan showing an enhancing lesion in left side larynx and pyriform sinus with erosion of thyroid cartilage and critical glottis opening. (B) USGTC-FNAC obtained using a 25 gauge spinal needle (solid line) which has been introduced through eroded thyroid cartilage.

After selection of patient for USGTC-FNAC, the initial step was to evaluate the computed tomography images (Fig. 1A and B), to localise the mass and the potential needle track. Then a check ultrasound was performed using a 7.5 MHz linear transducer after positioning the patient during reclining and head extended. The skin was cleaned with antiseptic and covered with a sterile drape, and 3–5 mL of 2% lignocaine was injected using 25 gauge spinal needles. Before infiltrating the local anaesthetic, ultrasound (US) images of the lesions was obtained. Computed tomography images obtained during the preoperative work up were studied and lesions approached accordingly through the thyrohyoid, cricothyroid membrane or thyroid cartilage, if it was eroded by tumour as shown in Fig. 1A and B.

After locating the site, the needle was advanced under US guidance until it reached the interior of the lesion (Fig. 1B). A drop of the aspirate was placed on glass slides, which was later placed in 10% phosphate buffered formalin, followed by standard (H & E) haematoxylin and eosin staining. At the end of procedure, patients were kept under observation for 30 min and then discharged.

## Results

A total of 24 patients underwent USGTC-FNAC (male, 21; female, 3) for laryngo-hypopharyngeal masses in the age group of 40–73 years with the mean age of 56.7 years. Of these 24 patients, 23 patients had suspicious untreated mass lesions obstructing the laryngeal lumen, and one patient had received laser cordectomy for glottis cancer T_1_N_0_ M_0_ (AJCC 8th edition) (Table 1).

**Table 1:** Patients characteristic.

No.	Age/gender	Lesion site	Clinical diagnosis	Previous treatment	USGTC-FNAC	TNM
1	55/F	Supraglottis	Malignancy	Nil	SCC	T3 N2c M0
2	73/M	Transglottic	Malignancy	Nil	PDSCC	T4a N0 M0
3	55/M	Supraglottis	Malignancy	Nil	SCC	T4a N2b M0
4	45/M	Transglottic	Malignancy	Laser resection	SCC	T4a N2b M0
5	68/M	Transglottic	Malignancy	Nil	SCC	T4b N0 M0
6	40/M	Supraglottis	Malignancy	Nil	Atypical cells	T3 N1 M0
7	53/M	Supraglottis	Malignancy	Nil	PDSCC	T4a N2c M0
8	45/F	Transglottic	Malignancy	Nil	SCC	T3 N0 M0
9	65/M	Laryngopharynx	Malignancy	Nil	Inadequate sample	T3 N0 M1
10	50/M	Laryngopharynx	Malignancy	Nil	SCC	T4a N2b M0
11	40/M	Supraglottis	Malignancy	Nil	SCC	T4a N0 M0
12	60/M	Supraglottis	Malignancy	Nil	SCC	T4a N1 M0
13	71/M	Laryngopharynx	Malignancy	Nil	SCC	T3 N1 M0
14	58/M	Supraglottis	Malignancy	Nil	PDSCC	T3 N1 M0
15	58/M	Laryngopharynx	Malignancy	Nil	Atypical cells	T3 N1 M0
16	65/M	Supraglottis	Malignancy	Nil	SCC	T3 N1 M0
17	50/M	Transglottic	Malignancy	Nil	SCC	T3 N0 M0
18	72/M	Supraglottis	Malignancy	Nil	SCC	T3 N3 B M0
19	67/M	Base of tongue	Malignancy	Nil	SCC	T4a N0 M0
20	50/F	Transglottic	Malignancy	Nil	SCC	T4a N1 M0
21	50/M	Transglottic	Malignancy	Nil	SCC	T4b N0 M0
22	60/M	Laryngopharynx	Malignancy	Nil	SCC	T3 N0 M0
23	55/M	Laryngopharynx	Malignancy	Nil	SCC	T4a N0 M0
24	56/M	Transglottic	Malignancy	Nil	SCC	T4 N0 M0

M, male; SCC, squamous cell carcinoma; PDSCC, poorly differentiated squamous cell carcinoma; T, tumour; N, node; M, metastasis; USGTC-FNAC, Ultrasound Guided Trans Cutaneous Fine Needle Aspiration Cytology.

The tumour was localised without any difficulty by ultrasound in all 24 patients. Ultrasound-guided fine needle aspiration was undertaken in all. Since all patients had advanced (T_3_/T_4_) disease we did not experience any technical difficulty either in localising the lesion or obtaining the tissue for diagnosis. All patients tolerated the procedure well without any complications or technical problems.

Cytology evaluation revealed squamous carcinoma in 21 cases (87.5%) and 2 cases (8%) atypical cells, and the remaining biopsy contained an inadequate sample (4%). Those three patients with equivocal/negative tests underwent direct laryngoscopy and were confirmed to have squamous carcinoma on biopsy.

## Discussion

Our experience of USGTC-FNAC in 24 patients with bulky laryngeal pharyngeal lesions suggests that this technique was feasible and quite sufficient for diagnosis in 21 of 24 cases with diagnostic sensitivity of 87.5%.

Dedivitis et al. studied 28 patients with transcutaneous fine needle aspiration biopsy of the pre-epiglottic space who underwent partial laryngectomy for supraglottic squamous cell carcinoma and compared that with histopathologic analysis of the laryngectomy specimens. Efficiency was found to be in range of 96.4%, without any morbidity.^5^

Ansarin et al. performed Ultrasound-Guided Transcutaneous Tru-Cut Biopsy in ten patients: 4 were treatment naïve had mass lesion obstructing the laryngeal lumen and 6 were previously treated for laryngeal cancer. Out of these 10 patients, 9 patients had adequate tissue and diagnosed with 100 percent sensitivity on cytology.^6^

Preda et al. followed the same biopsy procedure in selected patients (with stenosis of airways or difficult intubation or contraindication to general anaesthesia) as Ansrin et al. and proved the sensitivity of this technique was 92.5%.^7^

Kohli et al. studied 25 patients of carcinoma larynx and laryngeal pharynx with fine needle aspiration cytology and exfoliative cytology and direct laryngoscopy biopsy. The positivity rate with FNAC was 80%.^8^

Aspiration cytology techniques in larynx lesions are quite sensitive across various studies with sensitivity ranging from 80% to 100%, which is comparable with our study, with no major complications.

With respect to feasibility and safety of USGTC-FNAC, it was performed successfully in all 24 patients, making this procedure a viable option for patients who are not fit for direct laryngoscopy biopsy under general anaesthesia. All procedures were performed under local anaesthesia without any technical problems or complications.

Only one patient had an inadequate specimen for making any definitive diagnosis, which could have been avoided if adequacy of the specimen was checked immediately post procedure. Major advantages of this technique include less invasiveness, lower cost, and performance under local anaesthesia on an outpatient basis. This avoids the need for general anaesthesia, the risks and costs associated with direct laryngoscopy like damage to teeth, and protective tracheostomy in case of airway compromise.

We did not have post-therapy (chemo-radiotherapy) patients in our study population to comment on the feasibility of performing the cytology in that subgroup of patients. Tru cut biopsy was tried in few studies with acceptable results.

Tumour cell implantation along the needle tract is one of the rare complications in needle aspirations of malignant lesions in head and neck. In our institute we rarely came across implantation metastasis in the past. A search of literature also reveals very few reports of implantation metastasis in thyroid, submandibular glands, and cervical nodes. Accurate estimate is difficult to ascertain in these rare conditions, since crude estimates are 0.00012% for FNAC.^9^, ^10^

Direct Laryngoscopy and biopsy under general anaesthesia is currently the “Gold Standard” technique for tumour evaluation and for tissue diagnosis. Tumour evaluation today can however be almost equally well undertaken with flexible endoscopy and sectional imaging. Further laryngoscopy under anaesthesia is however associated with a definite risk of precipitating airway compromise and tracheotomy in some situations. The technique of Ultrasound guided FNAC as reported here is noted to be feasible and reliable in patients with locally advanced (T_3_–T_4_) supraglottic cancer and offers an alternate and safe option for tissue diagnosis in such patients.

## Conclusion

USGTC-FNAC for advanced laryngeal lesions is a feasible option but not an alternative for direct laryngoscopy biopsy. In a select group of patients, USGTC-FNAC can be done as an out-patient procedure without any preparation, which will enable the clinician to make rapid diagnosis and treatment plan. In the era of organ conservation, rapid diagnosis with minimally invasive technique will go a long way in expediting decision- making, avoiding risks and costs associated with the standard direct laryngoscopy evaluation.

## Conflicts of interest

The authors declare no conflicts of interest.
